# Hot beverage intake and oesophageal cancer in the UK Biobank: prospective cohort study

**DOI:** 10.1038/s41416-025-02953-2

**Published:** 2025-02-19

**Authors:** Maki Inoue-Choi, Yesenia Ramirez, Caitlin O’Connell, Amy Berrington de Gonzalez, Sanford M. Dawsey, Christian C. Abnet, Neal D. Freedman, Erikka Loftfield

**Affiliations:** 1 Division of Cancer Epidemiology and Genetics, National Cancer Institute, 9609 Medical Center Dr, Bethesda, MD USA; 2 The Institute of Cancer Research, Clinical Cancer Epidemiology, London, UK

**Keywords:** Risk factors, Oesophageal cancer

## Abstract

**Background:**

Drinking maté, a type of tea consumed at a very hot temperature in South America has been considered as a risk factor for oesophageal squamous cell carcinoma (ESCC).

**Methods:**

We assessed daily intake and preferred temperature of hot beverages (tea and coffee) in relation to incident ESCC (*n* = 242) and adenocarcinoma (EAC; *n* = 710) among 454,796 adults in the UK Biobank. Hazard ratios (HRs) and 95% confidence intervals (CIs) were estimated using multivariable Cox proportional hazards regression.

**Results:**

Relative to non-drinkers and warm temperature drinkers (referent group), drinking 4–6 cups/d (HR, 1.97; 95% CI, 1.14–3.38) or more of hot temperature beverages was associated with higher risk of ESCC; HRs increased with increasing daily intake of hot temperature beverages (*P*-trend < 0.01). ESCC risk was still higher for those who drank very hot beverages; drinking ≤ 4 cups/d was associated with a 2.5-fold higher risk (HR, 2.52; 95% CI, 1.27–5.03), and risk increased with increasing daily intake of very hot temperature beverages (*P*-trend < 0.01). There was no clear association for EAC.

**Conclusions:**

Our findings provide new evidence that drinking hot or very hot beverages is a risk factor for ESCC in the UK where drinking hot tea and coffee is common.

## Introduction

Oesophageal cancer is the seventh most common cancer and the sixth leading cause of cancer death, with more than 500,000 deaths (6% of all cancer deaths) recorded in 2020 worldwide [1]. Incidence and mortality of oesophageal cancer vary by region and culture with high incidence observed in East to Central Asia and Southern Africa. There are two main forms of oesophageal cancer: oesophageal squamous cell carcinoma (ESCC) and oesophageal adenocarcinoma (EAC), each with different established and suspected risk factors that vary in terms of population-attributable risk across populations. For ESCC, risk factors with consistent evidence include poverty, heavy alcohol consumption, tobacco smoking, and poor oral health, whereas for EAC risk factors include Barrett’s oesophagus, gastroesophageal reflux disease (GERD), and obesity as well as tobacco smoking [2–4].

Growing evidence indicates that thermal injury from drinking hot beverages is also a risk factor for ESCC. Drinking hot beverages, especially maté, a type of tea traditionally consumed in South America at very hot temperatures (≥65 °C = 149 °F), was classified as “probably carcinogenic to humans (Group 2 A)” by the International Agency for Research on Cancer (IARC), based on limited evidence for oesophageal cancer [5]. Evidence came mainly from hospital-based case-control studies in South America. Pooled analyses of studies conducted in South America found that higher intake of maté and other very hot beverages was associated with an increased risk of oesophageal cancer, but the associations were not observed for drinking beverages at lower temperatures [6, 7]. Similarly, case-control studies in Asia, the Middle East, and Africa and one prospective cohort study in Iran have found that drinking very hot or hot tea, compared with drinking tea at lower temperatures is associated with a higher risk of esophagael cancer, namely ESCC [8, 9]. Despite the worldwide popularity of hot beverages, studies of the association between beverage temperature and oesophageal cancer risk in Western populations are lacking; one small case-control study of 167 ESCC cases in Sweden found no association between drinking very hot beverages and oesophageal cancer highlighting the need for larger, prospective studies [10].

Herein, we evaluated the associations of hot beverage temperature and intake level with the incidence of oesophageal cancer in a large prospective cohort study of nearly half a million adults in the United Kingdom (UK), where hot beverages, namely tea and coffee, are commonly consumed.

## Methods

### UK Biobank

The UK Biobank is a large prospective cohort study of half a million adults who reside in the UK. The study design has been previously described in detail [11]. In brief, approximately 9.2 million adults aged 40 to 69 years who were registered with the UK National Health Service (NHS) and resided within 40 km of one of 22 NHS assessment centres across the UK (England, Wales, and Scotland) were invited to participate, of whom about half a million people electronically signed informed consent and completed touchscreen baseline questionnaires asking about sociodemographic, lifestyle, and health-related factors at an assessment centre between 2006 and 2010.

Of the 502,357 cohort members, we excluded participants with missing or incomplete data on tea intake (*n* = 2192) or coffee intake (*n* = 785), those who were pregnant (*n* = 368), those who did not drink tea or coffee but reported their preferred hot beverage temperature (*n* = 6042), those with missing or incomplete smoking data (*n* = 1884), those with registry confirmed prevalent cancer (except for non-melanoma skin cancer) (*n* = 26,441) at baseline, those who self-reported prevalent cancer on the baseline questionnaire (*n* = 9488), and those who reported tea or coffee drinking but not preferred hot beverage temperature (*n* = 361). After the exclusions, our analytic cohort consists of 454,796 participants.

### Hot beverage intake and covariate

Participants provided information about their usual diet, including hot beverage intake, via the touchscreen baseline questionnaire. Specifically, participants reported the number of cups of tea or coffee they drank per day. To avoid typing mistakes, participants who entered drinking > 20 cups of tea or > 10 cups of coffee were asked to re-type their response. Participants were also asked their preferred temperature of hot beverages, such as tea and coffee, by selecting from “very hot”, “hot”, “warm”, or “do not drink hot drinks”. We excluded 6042 participants who did not report drinking tea or coffee but did report their preferred hot beverage temperature as very hot, hot, or warm. We calculated total hot beverage intake as a sum of cups of tea and coffee consumed per day. We categorised participants who drank hot beverages according to preferred hot beverage temperature (hot or very hot) and daily intake levels of total hot beverages (≤ 4, > 4–6, > 6–8, and > 8 cups/d). Participants who reported drinking neither tea nor coffee and those who reported drinking tea or coffee at warm temperature were combined as the referent group.

Detailed information about tea and coffee drinking, such as the type of tea and coffee consumed and the use of milk or sugar additives, was collected in a series of 24-hour dietary recall questionnaires including one completed by a subset of 70,699 participants who were recruited in 2009 and 2010 and four additional questionnaires sent by e-mail to the entire cohort between 2011 and 2015. In the current study, a subset of 192,249 participants completed at least one 24-hour dietary recall questionnaire.

At baseline, participants provided information on potential confounders, including demographics (age, sex, race and ethnicity, and education), perceived general health (excellent, good, fair, and poor), and lifestyle (tobacco smoking, alcohol intake, physical activity, and dietary intake). We created a variable for physical activity as a sum of the number of days when participants had > 10 minutes of moderate or vigorous activity per week. Participants were asked about medical conditions including gastric reflux/GERD or esophagitis/Barrett’s oesophagus, by verbal interviews following the touchscreen questionnaire. Body mass index (BMI) was computed using height and weight measured at the initial physical assessment centre visit. We used the Townsend Deprivation Index, which is an area-level measure of material deprivation and factors in employment, car ownership, home ownership, and household crowding, to account socioeconomic status [12]. For tobacco smoking, we created a detailed 25-level variable by combining data on current and past smoking, type of tobacco currently smoked, type of tobacco previously smoked, number of cigarettes smoked per day, number of cigarettes previously smoked per day, and age at which they stopped smoking (for former smokers) as previously described [13]. For alcohol intake, we created a 6-level variable by combining alcohol intake status (never, former, and current) and, among current alcohol drinkers, the number of alcohol drinks consumed on a typical drinking day (never; former; current, < 1 drink/week; current, > 1- < 7 drinks/week; current, 1–3 drinks/d; and current, > 3 drinks/d). For dietary intake, we created variables for the daily intake of major food groups including fruits (fresh and dried; pieces/d), vegetables (cooked and raw; tablespoons/d), red meat (beef, lamb, and pork combined; servings/week), and processed meat (times/week).

### Incident cancer ascertainment

Cancer incidence was ascertained via linkage to national cancer registries [14]. All incident oesophageal cancer cases diagnosed through December 31, 2020 were identified based on the International Classification of Diseases, Tenth Revision code (ICD-10: C15) and categorised into two subtypes based on histology codes, including EAC (histology code: 8140, 8142, 8144, 8145, 8260, 8261, 8263, 8310, 8480, 8481, 8482, 8490, and 8570) and ESCC (histology code: 8070, 8071, 8072, and 8074).

### Statistical analysis

We tabulated demographic, lifestyle, medical, and dietary factors by hot beverage temperature and intake categories. Person-years were computed from the date of enrolment to the date of diagnosis of upper gastrointestinal cancer (oesophageal, gastric, or head and neck cancer), death, or the end of follow-up on December 31, 2020, whichever occurred first. Demographic, lifestyle, and medical characteristics were compared across the combined categories of hot beverage temperate and intake (≤ and > median = 6 cups/d). Because the type of tea or coffee and the addition of milk could affect hot beverage temperature, we assessed these factors by preferred beverage temperature and intake in a subset of 192,249 participants who completed at least one 24-hour dietary recall questionnaire. We computed hazard ratios (HRs) and 95% confidence intervals (CIs) for EAC or ESCC across the nine levels of combined categories of beverage temperature and intake level using Cox proportional hazards regression models adjusted for potential confounders including age, sex, race, education level, TDI, perceived general health, BMI, smoking status, physical activity level, vegetable intake, fruit intake, red meat intake, and processed meat intake. We also adjusted the final model for the type of hot beverages consumed (tea and coffee, tea only, coffee only, and neither tea nor coffee) to assess the association of tea temperature and intake with each cancer outcome independent of beverage type. Models for EAC were additionally adjusted for a history of GERD/gastric reflux and a history of esophagitis/Barrett’s oesophagus, which was also considered as a potential mediator. Models for ESCC were additionally adjusted for alcohol drinking status (Supplementary Fig. 1). The baseline risk was stratified by NHS assessment centre region (England, Scotland, and Wales). For missing data which occurred in less than 8% of the study participants for any single covariate, we included indicator variables representing missing categories in the regression models. We tested the proportional hazards assumption by creating Schoenfeld residual plots and found a minimal deviation from a horizontal line for both EAC and ESCC, indicating that the assumption held. To test linearity for the association between tea and coffee intake and incident EAC or ESCC, we created a continuous-scale variable by using median values of total hot beverage intake categories for hot and very hot beverage temperature groups, separately. To minimise potential reverse causality, we performed a lag analysis excluding the first two years of follow-up including 108 incident oesophageal cancer cases (83 EAC and 25 ESCC) which occurred during these two years. All analyses were performed using SAS version 9.4 (SAS Institute, Cary NC). There was no pre-determined power estimate because this study is a secondary data analysis within the existing cohort study.

## Results

Among 454,796 study participants, 53.2% (*n* = 242,177) were women and 46.8% (*n* = 242,619) were men; average age at enrolment was 56.9 (standard deviation, SD: 8.1; range: 38.9–73.7). Within the cohort, 99.0% of participants reported drinking either tea or coffee regularly with 20.1% drinking only tea, 12.6% drinking only coffee, and 66.3% drinking both tea and coffee. Among 450,050 participants who reported drinking tea and/or coffee, 66.5% preferred a hot temperature, 17.1% preferred a very hot temperature, and 15.3% preferred a warm temperature. Participants who reported drinking tea or coffee at hot temperatures were more likely to have good or excellent general health, have normal body weight, and be current alcohol drinkers compared with non- and warm-temperature drinkers. Participants who preferred drinking tea or coffee at very hot temperatures were more likely to be female and reported excellent general health and normal body weight. Those who reported drinking > 6 cups/d of hot beverages tended to be white individuals, have less education, and be current smokers or current alcohol drinkers compared with those who drank ≤ 6 cups/d (Table 1).

**Table 1:** Baseline demographic and lifestyle characteristics by preferred temperature and daily intake of hot beverages (tea or coffee).

	All participants	Tea or coffee drinking				
		Non-drinkers or warm temperature^a^	Hot temperature		Very hot temperature	
			≤6 cups/d^b^	>6 cups/d	≤6 cups/d	>6 cups/d
*N* (row %)	454,796 (100)	74,547 (16.4)	168,848 (37.1)	133,794 (29.4)	38,192 (8.4)	39,415 (8.7)
Age, mean (SD)	56.9 (8.1)	56.3 (8.3)	56.9 (8.2)	57.5 (7.8)	55.9 (8.2)	56.4 (7.9)
Sex (%)						
Female	242,177 (53.2)	38,968 (52.3)	91,010 (53.9)	66,295 (49.6)	23,562 (61.7)	22,342 (56.7)
Male	212,619 (46.8)	35,579 (47.7)	77,838 (46.1)	67,499 (50.5)	14,630 (38.3)	17,073 (43.3)
Race (%)^c^						
Asian	10,458 (2.3)	2607 (3.5)	5098 (3.0)	1125 (0.8)	1298 (3.4)	330 (0.8)
Black	7045 (1.5)	1192 (1.6)	3408 (2.0)	728 (0.5)	1382 (3.6)	335 (0.9)
White	428,936 (94.3)	68,940 (92.5)	156,876 (92.9)	130,364 (97.4)	34,568 (90.5)	38,188 (96.9)
Mixed	2677 (0.6)	494 (0.7)	1110 (0.7)	546 (0.4)	301 (0.8)	226 (0.6)
Other	4117 (0.9)	958 (1.3)	1791 (1.1)	645 (0.5)	509 (1.3)	214 (0.5)
Education (%)^c^						
College or University Degree	147,322 (32.4)	25,479 (34.2)	57,199 (33.9)	41,122 (30.7)	12,099 (31.7)	11,423 (29.0)
A/AS levels or equivalent	50,536 (11.1)	8702 (11.7)	19,328 (11.4)	14,144 (10.6)	4190 (11.0)	4172 (10.6)
O level or equivalent	95,646 (21.0)	14,881 (20.0)	35,702 (21.1)	28,042 (21.0)	8499 (22.3)	8522 (21.6)
CSEs or equivalent	24,833 (5.5)	3762 (5.0)	8919 (5.3)	7424 (5.6)	2317 (6.1)	2411 (6.1)
NVQ/HND/HNC equivalent	29,822 (6.6)	4804 (6.4)	10,236 (6.1)	9649 (7.2)	2361 (6.2)	2772 (7.0)
Other Professionalri Qualifications	23,147 (5.1)	3643 (4.9)	8364 (5.0)	7222 (5.4)	1866 (4.9)	2052 (5.2)
No formal education	75,466 (16.6)	11,778 (15.8)	26,129 (15.5)	23,942 (17.9)	6184 (16.2)	7433 (18.9)
Townsend deprivation index, mean (SD)	−1.32 (3.08)	−1.07 (3.21)	−1.33 (3.07)	−1.48 (2.99)	−1.18 (3.16)	−1.34 (3.07)
Assessment centre region						
Scotland	32,649 (7.2)	5519 (7.4)	13,242 (7.8)	8067 (6.0)	3255 (8.5)	2566 (6.5)
England	403,250 (88.7)	65,923 (88.4)	148,821 (88.1)	120,214 (89.9)	33,295 (87.2)	34,997 (88.8)
Wales	18,897 (4.2)	3105 (4.2)	6785 (4.0)	5513 (4.1)	1642 (4.3)	1852 (4.7)
BMI (%)^c^						
<18.5	2282 (0.5)	315 (0.4)	847 (0.5)	554 (0.4)	298 (0.8)	268 (0.7)
18.5 to <25	146,860 (32.3)	20,424 (27.4)	57,490 (34.0)	40,987 (30.6)	14,461 (37.9)	13,488 (34.2)
25 to <30	193,076 (42.5)	31,123 (41.7%)	71,139 (42.1)	59,206 (44.3)	14,984 (39.2)	16,624 (42.2)
≥30	110,183 (24.2)	22,168 (29.7)	38,477 (22.8)	32,462 (24.3)	8231 (21.6)	8845 (22.4)
Smoking (%)^d^						
Never	249,746 (54.9)	40,403 (54.2)	96,973 (57.4)	68,670 (51.3)	22,771 (59.6)	20,929 (53.1)
Former	156,618 (34.4)	25,160 (33.8)	58,555 (34.7)	47,421 (35.4)	12,415 (32.5)	13,067 (33.2)
Current	48,432 (10.6)	8984 (12.1)	13,320 (7.9)	17,703 (13.2)	3006 (7.9)	5419 (13.7)
Alcohol intake (%)^c^						
Never	19,341 (4.3)	4409 (5.9)	7122 (4.2)	4230 (3.2)	2095 (5.5)	1485 (3.8)
Former	15,563 (3.4)	3347 (4.5)	4811 (2.9)	4691 (3.5)	1191 (3.1)	1523 (3.9)
Current, <1 drink/week	101,813 (22.4)	19,240 (25.8)	35,113 (20.8)	30,176 (22.6)	8173 (21.4)	9111 (23.1)
Current, > 1 to <7 drinks/week	113,307 (24.9)	17,343 (23.3)	43,616 (25.8)	33,472 (25.0)	9471 (24.8)	9405 (23.9)
Current, 1 to 3 drinks/day	165,315 (36.3)	23,764 (31.9)	62,948 (37.3)	50,287 (37.6)	13,916 (36.4)	14,400 (36.5)
Current, >3 drinks/day	38,916 (8.6)	6339 (8.5)	15,037 (8.9)	10,786 (8.1)	3305 (8.7)	3449 (8.8)
Physical activity (days per week) (%)^c,e^						
0	47,362 (10.4)	8649 (11.6)	16,558 (9.8)	13,571 (10.1)	4103 (10.7)	4481 (11.4)
1–2	57,888 (12.7)	9687 (13.0)	21,647 (12.8)	16,994 (12.7)	4757 (12.5)	4803 (12.2)
3–4	74,622 (16.4)	11,872 (15.9)	28,563 (16.9)	22,003 (16.4)	6070 (15.9)	6114 (15.5)
≥5	238,844 (52.5)	38,161 (51.2)	88,878 (52.6)	70,402 (52.6)	20,323 (53.2)	21,080 (53.5)
Perceived general health (%)^c^						
Excellent	76,641 (16.9)	10,826 (14.5)	29,179 (17.3)	20,958 (15.7)	8021 (21.0)	7657 (19.4)
Good	264,291 (58.1)	41,317 (55.4)	100,404 (59.5)	79,221 (59.2)	21,435 (56.1)	21,914 (55.6)
Fair	92,914 (20.4)	17,717 (23.8)	32,664 (19.3)	27,710 (20.7)	6987 (18.3)	7836 (19.9)
Poor	19,028 (4.2)	4266 (5.7)	5894 (3.5)	5403 (4.0)	1605 (4.2)	1860 (4.7)
Gastric reflux/GERD (%)	18,813 (4.1)	3369 (4.5)	6570 (3.9)	5888 (4.4)	1425 (3.7)	1561 (4.0)
Esophagitis/Barrett’s oesophagus (%)	1322 (0.3)	255 (0.3)	454 (0.3)	429 (0.3)	84 (0.2)	100 (0.3)
**Dietary intake**						
Hot beverage type (%)						
Tea and coffee	301,570 (66.3)	42,709 (57.3)	102,324 (60.6)	104,930 (78.4)	22,115 (57.9)	29,492 (74.8)
Tea only	91,402 (20.1)	15,560 (20.9)	37,953 (22.5)	20,683 (15.5)	9815 (25.7)	7391 (18.8)
Coffee only	57,078 (12.6)	11,532 (15.5)	28,571 (16.9)	8181 (6.1)	6262 (16.4)	2532 (6.4)
Neither tea nor coffee	4746 (1.0)	4746 (6.4)	0 (0.0)	0 (0.0)	0 (0.0)	0 (0.0)
Coffee (cups/day), mean (SD)	2.1 (2.1)	1.9 (2.1)	1.5 (1.4)	2.7 (2.5)	1.5 (1.4)	2.7 (2.7)
Tea (cups/day), mean (SD)	3.5 (2.9)	2.9 (2.9)	2.2 (1.6)	5.1 (3.1)	2.3 (1.6)	5.5 (3.6)
Vegetables (tablespoons/day), mean (SD)	4.9 (3.4)	5.0 (3.7)	4.9 (3.3)	4.9 (3.2)	4.9 (3.4)	4.9 (3.4)
Fruits (pieces/day), mean (SD)	3.1 (2.6)	3.1 (2.7)	3.1 (2.5)	3.0 (2.5)	3.1 (2.7)	3.0 (2.7)
Red meat (servings/week), mean (SD)	2.1 (1.5)	2.1 (1.5)	2.1 (1.4)	2.2 (1.5)	2.1 (1.5)	2.2 (1.5)
Processed meat (times/week) (%)^c^						
0	41,309 (9.1)	7684 (10.3)	15,959 (9.5)	9830 (7.3)	4376 (11.5)	3460 (8.8)
<1 time/week	137,305 (30.2)	22,284 (29.9)	53,621 (31.8)	37,434 (28.0)	12,605 (33.0)	11,361 (28.8)
1 time/week	132,870 (29.2)	20,681 (27.7)	49,517 (29.3)	40,463 (30.2)	10,690 (28.0)	11,519 (29.2)
2 to 4 times/week	124,211 (27.3)	20,363 (27.3)	43,519 (25.8)	40,016 (29.9)	9109 (23.9)	11,204 (28.4)
5 to 6 times/week	14,478 (3.2%)	2571 (3.5)	4700 (2.8)	4729 (3.5)	1034 (2.7)	1444 (3.7)
≥7 times/week	3743 (0.8%)	769 (1.0)	1175 (0.7)	1132 (0.9)	288 (0.8)	379 (1.0)

*BMI* body mass index, *GERD* gastroesophageal reflux disease, *SD* standard deviation.

^a^Participants who reported not drinking tea or coffee (*n* = 4746) and those who reported drinking warm beverages (*n* = 69,801) were combined.

^b^Median intake of total hot beverages (tea and coffee).

^c^Percentages were computed including participants with missing/unknown data and therefore may not equal 100%.

^d^Detailed 25-level variable has been described previously (ref).

^e^Number of days when participants had more than 10 minutes of moderate or vigorous activity per day.

Tea and coffee drinking patterns, such as types of tea and coffee and addition of milk, are shown by hot beverage temperature and intake level in a subset of participants who completed at least one 24-hour dietary questionnaire (Supplementary Table 1**)**. Of these 192,249 individuals, 80.4% reported drinking tea and 71.7% reported drinking coffee. Compared to those who drank ≤ 6 cups/d (median) of tea or coffee, higher proportions of those who drank > 6 cups/d of tea or coffee (89.0% hot and 88.8% very hot temperature) drank tea, and lower proportions of them (76.3% hot and 73.4% very hot temperature) drank coffee. Among tea drinkers, 88.5% drank black tea, and more than 90% of those who drank > 6 cups/d of tea or coffee drank black tea. About 90% of black tea drinkers reported adding milk to their tea across hot beverage temperature and intake level groups. The most popular type of coffee consumed was instant coffee (68.3%), followed by filtered coffee (30.6%). Compared with those who drank ≤ 6 cups/d of tea or coffee, more individuals who drank > 6 cups/d of tea or coffee (55.9% hot and 54.2% very hot temperature) drank instant coffee. There was no clear pattern in addition of milk to instant or filtered coffee across beverage temperature and intake level groups.

During an average follow-up of 11.6 years (5,292,347 total person-years), we identified 710 EAC and 242 ESCC. For our analysis of beverage temperature, we used a referent group that included participants who did not drink coffee or tea and those who preferred drinking these beverages at a warm temperature. Relative to this referent group, participants who preferred beverages at hot temperature and drank > 4 cups/d of tea or coffee had a higher risk of ESCC independent of beverage type consumed; the HRs (95% CI) were 1.62 (0.94–2.81) for ≤ 4 cups/d, 1.97 (1.14–3.38) for > 4 to 6 cups/d, 2.48 (1.38–4.44) for > 6 to 8 cups/d, and 3.10 (1.69–5.71) for > 8 cups/d (*P*-trend <0.01) (Table 2). Participants who preferred very hot beverages had still higher risks independent of beverage type; the HRs (95% CI) were 2.52 (1.27–5.03) for ≤ 4 cups/d, 3.67 (1.96–6.86) for > 4-6 cups/d, 4.75 (2.44–9.26) for > 6-8 cups/d, and 5.64 (2.88-11.00) for > 8 cups/d (*P*-trend <0.01). For EAC, HRs across intake levels, including > 8 cups/d, were not statistically significant nor did they show a clear pattern of higher risk with higher temperature and intake level. Removing GERD and Barrett’s oesophagus, which could be on the causal pathway, from the EAC model did not meaningfully alter HR estimates (Supplementary Table 2). Excluding the first two years of follow-up, similar associations were observed, with slightly stronger HRs for most intake levels of hot or very hot preferred beverage temperatures (Table 2).

**Table 2:** Risk of oesophageal squamous cell carcinoma and adenocarcinoma by preferred temperature and daily intake level of hot beverages (tea and coffee).

	Oesophageal adenocarcinoma				Oesophageal squamous cell carcinoma			
	*N*	Case N	HR (95% CI)^a^	*P*-trend^b^	*N*	Case N	HR (95% CI)^c^	*P*-trend
**All participants**	454,796	710			454,796	242		
Participant *N*								
Non-drinkers or warm temperature^d^	74,547	127	1.00 (reference)		74,547	18	1.00 (reference)	
Hot								
≤ 4 cups/d	110,344	120	0.70 (0.54–0.90)	0.02	110,344	45	1.62 (0.94–2.81)	<0.01
> 4–6 cups/d	106,088	165	0.90 (0.71–1.14)		106,088	50	1.97 (1.14–3.38)	
> 6–8 cups/d	54,514	104	1.05 (0.81–1.37)		54,514	32	2.48 (1.38–4.44)	
> 8 cups/d	31,696	87	1.31 (0.99–1.73)		31,696	26	3.10 (1.69–5.71)	
Very hot								
≤ 4 cups/d	24,226	21	0.68 (0.43–1.08)	0.90	24,226	15	2.52 (1.27–5.03)	<0.01
> 4–6 cups/d	26,132	45	1.21 (0.86–1.70)		26,132	22	3.67 (1.96–6.86)	
> 6–8 cups/d	15,777	23	0.97 (0.62–1.51)		15,777	17	4.75 (2.44–9.26)	
> 8 cups/d	11,472	18	0.92 (0.56–1.51)		11,472	17	5.64 (2.88–11.00)	
**Lag analysis excluding first 2 years of follow-up**								
Participant N	452,738	627			452,738	217		
Non-drinkers or warm temperature	74,182	114	1.00 (reference)		74,182	15	1.00 (reference)	
Hot								
≤4 cups/d	109,870	103	0.67 (0.51–0.88)	0.03	109,870	43	1.84 (1.02–3.32)	<0.01
>4–6 cups/d	105,635	143	0.88 (0.68–1.13)		105,635	43	2.01 (1.12–3.64)	
>6–8 cups/d	54,266	92	1.04 (0.79–1.38)		54,266	29	2.68 (1.43–5.04)	
>8 cups/d	31,510	76	1.30 (0.96–1.74)		31,510	24	3.49 (1.81–6.70)	
Very hot								
≤4 cups/d	24,139	21	0.75 (0.47–1.20)	0.80	24,139	14	2.80 (1.35–5.83)	<0.01
>4–6 cups/d	26,028	40	1.21 (0.84–1.73)		26,028	19	3.78 (1.92–7.46)	
>6–8 cups/d	15,695	22	1.04 (0.65–1.64)		15,695	13	4.34 (2.06–9.17)	
>8 cups/d	11,413	16	0.92 (0.54–1.56)		11,413	17	6.91 (3.42–13.95)	

^a^ Hazard ratio (HR) and 95% confidence interval (CI) adjusted for age, sex, race (white or other), Townsend Deprivation Index, general health status (excellent, good, fair, or poor), body mass index (<18, 18 to <25, 25 to <30, ≥30 kg/m2), tobacco smoking (25-levels including current smoking status, smoking intensity [current and former smokers], time since quitting [former smokers], and cigar and pipe use [current and former smokers]), physical activity (>10 minutes of moderate or vigorous activity; days per week), and dietary intake including vegetables (tablespoons per day), fruits (pieces per day), red meat (beef, lamb, and pork; times/week), and processed meat (0, <1, 1, 2 to 4, 5 to 6, and ≥7 times/week), history of gastroesophageal reflux disease (GERD)/gastric reflux (yes/no), and history of esophagitis/Barrett’s oesophagus (yes/no). The baseline risk was stratified by the UK National Health Service assessment centre region (England, Scotland, and Wales).

^b^ Tests for trend across intake categories in hot or very hot temperature group including the reference group (non-drinkers and warm temperature drinkers). A value zero was assigned for the reference group.

^c^ Regression models were adjusted for covariates listed above except for history of GERD/gastric reflux (yes/no) and history of esophagitis/Barrett’s oesophagus (yes/no), and additionally adjusted for alcohol intake (never drinker, former drinker, infrequent drinker [<1 drink/week], occasional drinker [>1 drink/week but <1 drink/day], moderate daily drinker [1 to 3 drink/day]), or heavy drinker [>3 drinks per day]).

^d^ Participants who reported not drinking tea or coffee (*n* = 4746) and those who reported drinking warm beverages (*n* = 69,801) served as the referent group.

## Discussion

Very hot beverages (> 65 °C) have been classified as “probably carcinogenic to humans (Group 2 A)” based largely on evidence from ESCC case-control studies of maté and tea consumption in South America, Iran, and Asia [5–9]. However, despite widespread consumption, it is unknown whether drinking very hot beverages is a risk factor for ESCC in other parts of the world, including in Europe and other Western populations. In this large prospective cohort study of almost half a million adults in the UK where hot tea and coffee, especially black tea and instant coffee, are widely consumed, we found that drinking higher amounts of very hot beverages was associated with a higher incidence of ESCC, but not EAC, in a dose-dependent manner and regardless of type of beverages consumed.

Strengths of the current study include the assessment of daily intake and preferred temperature of hot beverages, which allowed us to evaluate the relative risk of oesophageal cancer at increasing levels of intake and temperature. Furthermore, 99% of the UK Biobank study participants reported drinking tea or coffee, resulting in a wide range of intake and temperature preferences. Finally, although oesophageal cancer is a relatively uncommon cancer in the UK, the large cohort size, prospective design, and extended follow-up period resulted in a sufficient number of incident cases to evaluate associations for ESCC and EAC separately. There are certain limitations to be noted. We analysed self-reported tea and coffee intake and preferred hot beverage temperature at baseline, and therefore, changes in preference or intake during follow-up could have led to exposure misclassification. However, in a subset of cohort participants, daily intakes of tea and coffee reported on two touchscreen questionnaires administered four years apart were highly concordant (weighted κ = 0.83 for both) [15]. Reliability of self-reported hot tea temperature has been shown in previous studies with good concordance between self-reported chai tea temperature in two questionnaires in Kenya (weighted κ = 0.54) and two measurements using the validated method in Iran (weighted κ = 0.71) [16, 17]. In the study in Kenya, there was a moderate agreement between self-report and measured temperature (weighted κ = 0.46 and 0.51) [16]. Nevertheless, potential exposure misclassification would likely be nondifferential with respect to cancer incidence, and attenuate risk estimates, given the prospective cohort design. We adjusted Cox regression models for potential confounders, including known demographic, lifestyle, and medical factors for these cancers, but residual confounding by unmeasured or poorly measured factors is possible.

Drinking very hot beverages has been associated with elevated risk of oesophageal cancer in South America and Iran, where maté or tea are traditionally consumed at very hot temperatures (i.e., ≥ 65 °C) and where a majority of oesophageal cancer cases are ESCC [6–8]. Lower beverage temperatures, including hot and warm, are currently “not classified as carcinogenic to humans (Group 3)” by the IARC; however, studies evaluating lower qualitative beverage temperatures (i.e., <65 °C) are more limited [18]. A recent meta-analysis of eight case-control studies mostly from China and one each from Iran and Kenya showed that drinking very hot tea was associated with 1.94 (95% CI, 1.64–2.24) times higher risk of ESCC compared with drinking warm or no tea [19]. Other studies In China and Japan, where green tea drinking is common, have shown an association between drinking hot or very hot beverage and oesophageal cancer [20, 21]. Eating very hot food, such as hot nsima (corn meal; local staple food and commonly consumed very hot) in Malawi (odds ratio [OR], 2.8; 95% CI, 1.3-5.8) [22] and burning hot soup or porridge in China (OR, 4.75; 95% CI, 3.33-6.79 for men and OR, 6.77; 95% CI, 4.09–11.20 for women) [23] has also been associated with higher risk of ESCC. Unlike maté drinking, people in most regions of the world are thought to drink hot beverages at <65 °C. For example, coffee is thought to be typically consumed at around 60 °C (140 °F) in the U.S [16, 24]. In the UK Biobank, preferred temperature of hot beverages was self-reported as “warm”, “hot”, or “very hot” and temperature anchors (e.g., <65 °C) were not included in the questionnaire. However, previous, albeit a limited number of, studies in the UK have found that tea or coffee was generally consumed at between 54 and 62 °C, which is below the threshold of very hot beverages as cited by the IARC report [16]. In comparison, tea or maté was consumed at ≥ 70 °C in South America [16]. A majority of tea or coffee drinkers in the current study preferred a hot beverage temperature, which is likely < 65 °C. Furthermore, in this study, high proportions of hot beverage drinkers (90% of black tea and 83% of instant coffee, the two most drunken types of tea and coffee) reported adding milk to their tea or coffee, which could further lower tea temperature. Still a hot or very hot temperature preference was associated with a higher incidence of ESCC, which is consistent with findings from prior studies conducted on tea and maté in other parts of the world and with the conclusions of the IARC monograph [5].

Potential biologic mechanisms underlying the association of very hot beverage drinking with oesophageal cancer risk are unclear. One hypothesis is that habitual exposure to very hot beverages causes cell injuries that contribute to the development of cancer in the oesophagus. An animal study showed that ingesting very hot water (70 °C) had a tumour promoter effect on oesophageal hyperproliferative premalignant lesions in rats [25]. In another animal study, drinking maté in water at lower temperature than 65 °C did not promote existing DNA damages in oesophageal lesions in rats [26]. These findings indicate that the increased risk of oesophageal cancer among individuals who drank maté or other hot beverages at very hot temperatures was due to very hot beverage temperatures rather than maté or other beverage itself. Our results support this hypothesis, as they suggest that drinking tea and coffee at a high or very high temperature, like maté, is associated with ESCC. We observed clear associations for ESCC but not for EAC in our study. Although ESCC and EAC arise within the same organ, substantial prior work indicates that these subtypes have distinct incidence patterns, etiologies, and pathogenesis [2]. Our findings provide additional evidence in support of this prior evidence.

Although our findings suggest that drinking tea and coffee at hot and very hot temperatures is associated with increased risk of ESCC, it is important to make decisions about diet within the broader context of each beverage in health, rather than as a result of a single study or concerns about a single disease. Tea and coffee have each been associated inversely with overall mortality and incidence of a number of common diseases [13, 27–30]. However, individuals who like their beverages very hot might benefit from reducing the temperature of their beverages, at least with regards to their risk of ESCC.

In conclusion, we found that tea and coffee drinkers in the UK who preferred their beverages hot or very hot, had a higher incidence of ESCC relative to those who did not drink hot beverages or preferred to drink them warm. Additionally, we found no consistent evidence linking hot beverage temperature to incidence of EAC. Our findings extend what is known about the carcinogenicity of drinking hot and very hot beverages to an adult Western population where tea and coffee with addition of milk are consumed at high quantities.

## Supplementary information

Supplementary Tables and Figures

## Data Availability

The data underlying this article were provided by UK Biobank by proposal approval and are available from the UK Biobank on request (https://cprd.com/data-access).

## Code availability

The statistical code used to generate the results will not be available to access.
