# Supplementary material for: Hot beverage intake and oesophageal cancer in the UK Biobank: prospective cohort study
Source: Br J Cancer. 2025 Feb 19;132(7):652–9. doi: 10.1038/s41416-025-02953-2 (PMC11961563; doi:10.1038/s41416-025-02953-2)
Supplement: Supplementary file 1 — Supplementary Tables and Figures [file 41416_2025_2953_MOESM1_ESM.docx]

**SUPPLEMENTARY TABLES AND FIGURES**

**Supplementary Table 1. Tea and coffee drinking characteristics^a^ by preferred temperature and daily intake of hot beverages (tea and coffee) in a subset of 192,249 individuals who completed at least one 24-hour dietary recall questionnaires**

|  | **All participants** | **Tea or coffee drinking** | | | | |
| --- | --- | --- | --- | --- | --- | --- |
|  |  | **Non-drinkers or warm temperature^b^** | **Hot temperature** | | **Very hot temperature** | |
|  |  |  | **≤ 6 cups/d^c^** | **> 6 cups/d** | **≤ 6 cups/d** | **> 6 cups/d** |
| N (row %) | 192,249 (100) | 31,937 (16.6) | 73,311 (38.1) | 55,195 (28.7) | 16,121 (8.4) | 15,685 (8.2) |
| Tea drinking |  |  |  |  |  |  |
| No, n (%) | 37,734 (19.6) | 8,957 (28.1) | 17,386 (23.7) | 6,047 (11.0) | 3,591 (22.3) | 1,753 (11.2) |
| Yes, n (%) | 154,515 (80.4) | 22,980 (71.9) | 55,925 (76.3) | 49,148 (89.0) | 12,530 (77.7) | 13,932 (88.8) |
| Black, n (%)^d^ | 136,822 (88.5) | 19,380 (84.3) | 48,318 (86.4) | 45,436 (92.5) | 10,808 (86.3) | 12,880 (92.5) |
| Green, n (%) | 12,326 (8.0) | 2,268 (9.9) | 4,782 (8.6) | 3,388 (6.9) | 1,023 (8.2) | 865 (6.2) |
| Herbal, n (%) | 18,521 (12.0) | 3,225 (14.0) | 8,095 (14.5) | 4,291 (8.7) | 1,748 (14.0) | 1,162 (8.3) |
| Rooibos, n (%) | 7,685 (5.0) | 1,261 (5.5) | 2,961 (5.3) | 2,242 (4.6) | 603 (4.8) | 618 (4.4) |
| Other, n(%) | 6,268 (4.1) | 1,109 (4.8) | 2,290 (4.1) | 1,760 (3.6) | 551 (4.4) | 558 (4.0) |
| Milk additive use |  |  |  |  |  |  |
| Black, n (%)^e^ | 123,563 (90.3) | 17,119 (88.3) | 43,766 (90.6) | 41,478 (91.3) | 9,623 (89.0) | 11,577 (89.9) |
| Rooibos, n (%) | 3,827 (49.8) | 574 (45.5) | 1,436 (48.5) | 1,203 (53.7) | 290 (48.1) | 324 (52.4) |
| Coffee drinking |  |  |  |  |  |  |
| No | 54,334 (28.3) | 10,586 (33.2) | 21,338 (29.1) | 13,103 (23.7) | 5,136 (31.9) | 4,171 (26.6) |
| Yes | 137,925 (71.7) | 21,351 (66.8) | 51,973 (70.9) | 42,995 (76.3) | 9,985 (68.1) | 11,514 (73.4) |
| Filtered, n (%)^f^ | 42,239 (30.6) | 6,305 (19.7) | 17,250 (23.5) | 12,200 (22.1) | 3,366 (20.9) | 3,118 (19.9) |
| Instant, n (%) | 94,161 (68.3) | 14,723 (46.1) | 32,955 (45.0) | 30,874 (55.9) | 7,102 (44.1) | 8,507 (54.2) |
| Espresso, n (%) | 4,620 (3.4) | 821 (2.6) | 2,040 (2.8) | 1,132 (2.1) | 378 (2.3) | 249 (1.6) |
| Capuccino, n (%) | 10,822 (7.9) | 1,598 (5.0) | 4,497 (6.1) | 2,925 (5.3) | 939 (5.8) | 863 (5.5) |
| Latte, n (%) | 10,124 (7.3) | 1,498 (4.7) | 3,856 (5.3) | 2,931 (5.3) | 929 (5.8) | 910 (5.8) |
| Other, n (%) | 1,919 (1.4) | 337 (1.1) | 719 (1.0) | 552 (1.0) | 153 (1.0) | 158 (1.0) |
| Milk additive use |  |  |  |  |  |  |
| Filtered, n (%)^g^ | 28,562 (67.6) | 4,170 (66.1) | 11,586 (67.2) | 8,610 (70.6) | 2,110 (62.7) | 2,086 (66.9) |
| Instant, n (%) | 78,073 (82.9) | 11,856 (80.5) | 27,278 (82.8) | 26,209 (84.9) | 5,685 (80.1) | 7,045 (82.8) |
| Espresso, n (%) | 1,228 (26.6) | 209 (25.5) | 558 (27.4) | 287 (25.4) | 111 (29.4) | 63 (25.3) |
| Other^h^, n (%) | 1,132 (59.0) | 191 (56.7) | 417 (58.0) | 346 (62.7) | 79 (51.6) | 99 (62.7) |

^a^ Shown as n (%)

^b^ Participants who reported not drinking tea or coffee and those who reported drinking warm beverages were combined.

^c^ Median intake of total hot beverages (tea and coffee)

^d^ Percentage of drinkers of respective tea type among all tea drinkers. Percentages are not exclusive across tea types.

^e^ Percentage of individuals who reported adding milk among respective type of tea drinkers

^f^ Percentage of drinkers of respective coffee type among all tea drinkers. Percentages are not exclusive across coffee types.

^g^ Percentage of individuals who reported adding milk among respective type of coffee drinkers

^h^ Other type of coffee not including latte and cappuccino

**Supplementary Table 2. Risk of adenocarcinoma by preferred temperature and daily intake level of hot beverages (tea and coffee) without adjustment for GERD and Barrett’s Esophagus**

|  | **Esophageal adenocarcinoma** | | | |
| --- | --- | --- | --- | --- |
|  | **N** | **Case N** | **HR (95% CI)^a^** | ***P*-trend^b^** |
| **All participants** | 454,796 | 710 |  |  |
| Participant N |  |  |  |  |
| Non-drinkers or warm temperature^c^ | 74,547 | 127 | 1.00 (reference) |  |
| Hot |  |  |  |  |
| ≤ 4 cups/d | 110,344 | 120 | 0.69 (0.54 - 0.89) | 0.02 |
| > 4 – 6 cups/d | 106,088 | 165 | 0.90 (0.71 - 1.14) |  |
| > 6 – 8 cups/d | 54,514 | 104 | 1.05 (0.80 - 1.36) |  |
| > 8 cups/d | 31,696 | 87 | 1.31 (0.99 - 1.73) |  |
| Very hot |  |  |  |  |
| ≤ 4 cups/d | 24,226 | 21 | 0.68 (0.43 - 1.08) | 0.97 |
| > 4 – 6 cups/d | 26,132 | 45 | 1.20 (0.85 - 1.68) |  |
| > 6 – 8 cups/d | 15,777 | 23 | 0.96 (0.61 - 1.49) |  |
| > 8 cups/d | 11,472 | 18 | 0.90 (0.55 - 1.48) |  |

^a^ Hazard ratio (HR) and 95% confidence interval (CI) adjusted for age, sex, race (white or other), Townsend Deprivation Index, general health status (excellent, good, fair, or poor), body mass index (<18, 18 to <25, 25 to <30, ≥30 kg/m^2^), tobacco smoking (25-levels including current smoking status, smoking intensity [current and former smokers], time since quitting [former smokers], and cigar and pipe use [current and former smokers]); physical activity (>10 minutes of moderate or vigorous activity; days per week), and dietary intake including vegetables (tablespoons per day), fruits (pieces per day), red meat (beef, lamb, and pork; times/week), and processed meat (0, <1, 1, 2 to 4, 5 to 6, and ≥7 times/week). The baseline risk was stratified by the UK National Health Service assessment center region (England, Scotland, and Wales).

^b^ Tests for trend across intake categories in hot or very hot temperature group including the reference group (non-drinkers and warm temperature drinkers). A value zero was assigned for the reference group.

^c^ Participants who reported not drinking tea or coffee (n=4,746) and those who reported drinking warm beverages (n=69,801) served as the referent group.

**Supplementary Figure 1. Directed Acyclic Graph (DAG) of proposed causal pathway between hot beverage temperature and EAC (panel A) and ESCC (panel B)**

**A**


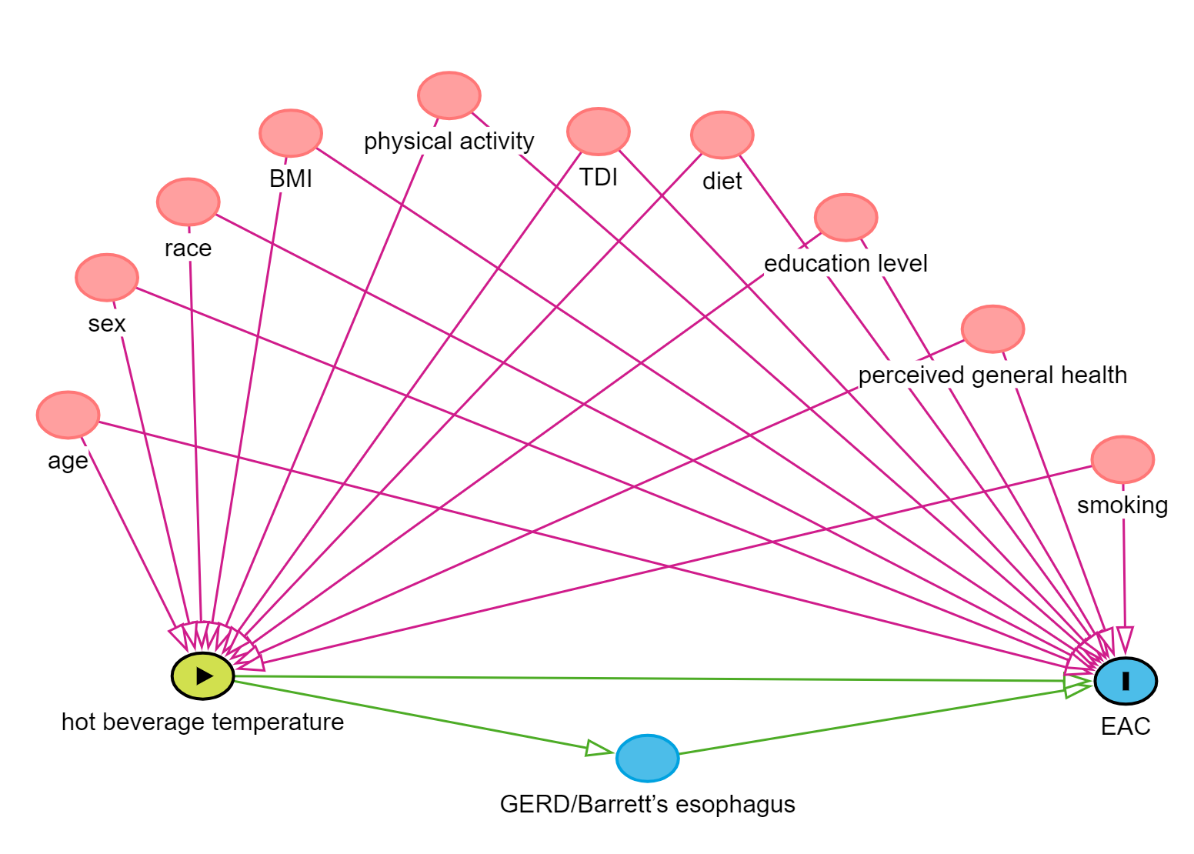


**B**


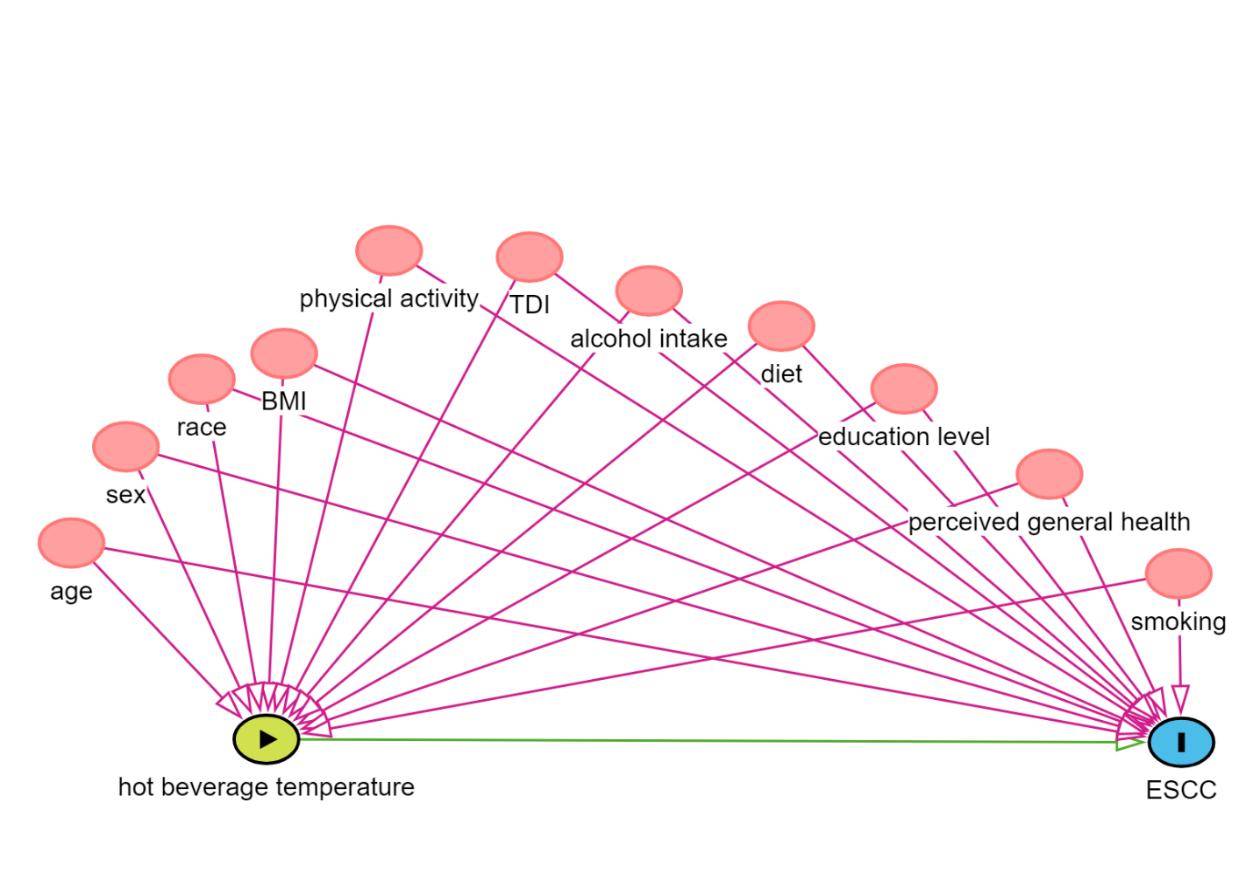


Abbreviations: BMI – Body mass index; TDI – Townsend Deprivation Index; GERD - gastroesophageal reflux disease ; EAC – Esophageal adenocarcinoma; ESCC – Esophageal squamous cell carcinoma
